# Supplementary material for: Incidence, Risk Factors and Clinical Implications of Glucose Metabolic Changes after Heart Transplant
Source: Biomedicines. 2022 Oct 26;10(11):2704. doi: 10.3390/biomedicines10112704 (PMC9687884; doi:10.3390/biomedicines10112704)
Supplement: Supplementary file 1 [file biomedicines-10-02704-s001.zip › biomedicines-1914802-supplementary.pdf]

**Table S1. Immune suppressive drug regimens in use in the 4 subgroups of patients divided according to glucose metabolic changes before and after transplant.**

| | Group A (n=23) | Group B (n=31) | Group C (n=36) | Group D (n=29) | p value |
|---|---|---|---|---|---|
| **Regimens** | | | | | 0.202 |
| CSA + RAD | 12 (52%) | 8 (25.8%) | 11 (30.5%) | 11 (37.9%) | |
| CSA + MPA | 8 (34.7%) | 11 (35.5%) | 11 (30.5%) | 13 (44.8%) | |
| FK + MPA | 2 (8.7%) | 11 (35.5%) | 13 (36.1%) | 4 (13.8%) | |
| RAD | 1 (4.4%) | 0 | 0 | 0 | |
| CSA | 0 | 1 (3.2%) | 1 (2.7%) | 1 (3.5%) | |

CSA, cyclosporin A; RAD, everolimus; MPA, mycophenolic acid; FK, tacrolimus.

**Table S2. Univariate and multivariable regression analysis of factors associated with mortality in the 119 patients studied.**

| | *Mortality* | | *Univariate analysis* | | | *Logistic regression* | | |
|---|---|---|---|---|---|---|---|---|
| | *Alive* | *Dead* | *Odds Ratio* | *(95% C.I.)* | *^p-value* | *Odds Ratio* | *(95% C.I.)* | *p-value* |
| ***Age:*** | | | | | | | | |
| ***≤58 years*** | *52* | *11* | *1.28* | *(0.51-3.20)* | *0.646* | | | |
| ***>58 years*** | *44* | *12* | | | | | | |
| ***Gender:*** | | | | | | | | |
| ***Male*** | *69* | *21* | *4.10* | *(0.90-18.7)* | *0.060* | *2.10* | *(0.41-10.7)* | *0.372* |
| ***Female*** | *27* | *2* | | | | | | |
| ***BMI pre-TX:*** | | | | | | | | |
| ***≤24.9*** | *51* | *10* | *1.47* | *(0.58-3.68)* | *0.489* | | | |
| ***>24.9*** | *45* | *13* | | | | | | |
| ***BMI post-TX:*** | | | | | | | | |
| ***≤25.2*** | *50* | *10* | *1.41* | *(0.56-3.53)* | *0.494* | | | |
| ***>25.2*** | *46* | *13* | | | | | | |
| ***Fasting Glyc. Pre-Tx:*** | | | | | | | | |
| ***≤101 mg/dL*** | *47* | *8* | *1.67* | *(0.62-4.50)* | *0.332* | | | |
| ***>101 mg/dL*** | *42* | *12* | | | | | | |
| ***Fasting Glyc. Post-Tx:*** | | | | | | | | |
| ***≤94 mg/dL*** | *52* | *7* | *2.19* | *(0.80-5.98)* | *0.144* | | | |
| ***>94 mg/dL*** | *44* | *13* | | | | | | |
| ***Gluc. Met. Profile Pre-Tx:*** | | | | | | | | |
| ***Normal*** | *43* | *4* | *3.85* | *(1.21-12.1)* | ***0.018*** | *4.25* | *(0.82-21.9)* | *0.084* |
| ***Hyperglycemic**** | *53* | *19* | | | | | | |
| ***Gluc. Met. Profile Post-Tx:*** | | | | | | | | |
| ***Normal*** | *56* | *10* | *1.82* | *(0.72-4.56)* | *0.245* | | | |
| ***Hyperglycemic**** | *40* | *13* | | | | | | |
| ***Total Cholesterol Pre-Tx:*** | | | | | | | | |
| ***≤153 mg/dL*** | *49* | *11* | *1.13* | *(0.45-2.89)* | *0.820* | | | |
| ***>153 mg/dL*** | *47* | *12* | | | | | | |
| ***Total Cholesterol Post-Tx:*** | | | | | | | | |
| ***≤184 mg/dL*** | *47* | *13* | *1.35* | *(0.54-3.38)* | *0.643* | | | |
| ***>184 mg/dL*** | *49* | *10* | | | | | | |
| ***HDL Cholesterol Pre-Tx:*** | | | | | | | | |
| ***≤43 mg/dL*** | *38* | *13* | *1.66* | *(0.62-4.47)* | *0.336* | | | |
| ***>43 mg/dL*** | *39* | *18* | | | | | | |
| ***HDL Cholesterol Post-Tx:*** | | | | | | | | |
| ***≤54.5 mg/dL*** | *45* | *10* | *2.22* | *(0.70-6.99)* | *0.266* | | | |
| ***>54.5 mg/dL*** | *50* | *5* | | | | | | |
| ***LDL Cholesterol Pre-Tx:*** | | | | | | | | |
| ***≤93 mg/dL*** | *39* | *11* | *1.07* | *(0.40-2.81)* | *1.000* | | | |
| ***>93 mg/dL*** | *38* | *10* | | | | | | |
| ***LDL Cholesterol Post-Tx:*** | | | | | | | | |
| ***≤100.5 mg/dL*** | *46* | *9* | *1.59* | *(0.52-4.84)* | *0.580* | | | |
| ***>100.5 mg/dL*** | *49* | *6* | | | | | | |
| ***Hypercholesterol. Pre-Tx:*** | | | | | | | | |
| ***No*** | *57* | *8* | *2.50* | *(0.94-6.62)* | *0.087* | *1.27* | *(0.42-3.84)* | *0.669* |
| ***Yes*** | *37* | *13* | | | | | | |
| ***Hypercholesterol. Post-Tx:*** | | | | | | | | |
| ***No*** | *38* | *13* | *1.98* | *(0.79-4.98)* | *0.164* | | | |
| ***Yes*** | *58* | *10* | | | | | | |

| | | | | | | | | |
|---|---|---|---|---|---|---|---|---|
| ***Triglycerides Pre-Tx:*** | | | | | | | | |
| ***≤95 mg/dL*** | *51* | *10* | *1.50* | *(0.60-3.37)* | *0.487* | | | |
| ***>95 mg/dL*** | *44* | *13* | | | | | | |
| ***Triglycerides Post-Tx:*** | | | | | | | | |
| ***≤151 mg/dL*** | *53* | *8* | *2.31* | *(0.89-5.965)* | *0.104* | | | |
| ***>151 mg/dL*** | *43* | *15* | | | | | | |
| ***Hypertriglycer. Pre-Tx:*** | | | | | | | | |
| ***No*** | *78* | *15* | *2.31* | *(0.85-6.29)* | *0.157* | | | |
| ***Yes*** | *18* | *8* | | | | | | |
| ***Hypertriglycer. Post-Tx:*** | | | | | | | | |
| ***No*** | *51* | *6* | *3.21* | *(1.16-8.84)* | ***0.022*** | *2.25* | *(0.74-6.79)* | *0.151* |
| ***Yes*** | *45* | *17* | | | | | | |
| ***Metabolic Synd Pre-Tx:*** | | | | | | | | |
| ***No*** | *76* | *12* | *3.48* | *(1.34-9.05)* | ***0.015*** | *1.85* | *(0.57-6.01)* | *0.304* |
| ***Yes*** | *20* | *11* | | | | | | |
| ***Metabolic Synd Post-Tx:*** | | | | | | | | |
| ***No*** | *74* | *15* | *1.79* | *(0.67-4.78)* | *0.286* | | | |
| ***Yes*** | *22* | *8* | | | | | | |
| ***eGFR Pre-Tx:*** | | | | | | | | |
| ***≤72.6 mL/min*** | *49* | *12* | *1.04* | *(0.42-2.60)* | *1.000* | | | |
| ***>72.6 mL/min*** | *47* | *11* | | | | | | |
| ***eGFR Post-Tx:*** | | | | | | | | |
| ***≤68.6 mL/min*** | *43* | *17* | *3.49* | *(1.26-9.62)* | ***0.019*** | *2.45* | *(0.80-7.48)* | *0.116* |
| ***>68.6 mL/min*** | *53* | *6* | | | | | | |

*^ p-value was generated by Fisher's exact test*

** Includes IFG/IGT and Diabetes Mellitus patients*

*For variables continuous to patients were divided on the basis of median values of each analyzed biomarker*
